# Discovery of a unique *Mycobacterium tuberculosis* protein through proteomic analysis of urine from patients with active tuberculosis

**DOI:** 10.1016/j.micinf.2017.12.011

**Published:** 2018-04

**Authors:** Nira Pollock, Rakesh Dhiman, Nada Daifalla, Maha Farhat, Antonio Campos-Neto

**Affiliations:** a Boston Children's Hospital and Harvard Medical School, Boston MA, USA; b The Forsyth Institute, Cambridge MA, USA; c Harvard Medical School, and Massachusetts General Hospital, Boston, MA, USA

**Keywords:** *Mycobacterium tuberculosis*, C strain, Antigenuria

## Abstract

Identification of pathogen-specific biomarkers present in patients' serum or urine samples can be a useful diagnostic approach. In efforts to discover *Mycobacterium tuberculosis* (*Mtb*) biomarkers we identified by mass spectroscopy a unique 21-mer *Mtb* peptide sequence (VVLGLTVPGGVELLPGVALPR) present in the urines of TB patients from Zimbabwe. This peptide has 100% sequence homology with the protein TBCG_03312 from the C strain of *Mtb* (a clinical isolate identified in New York, NY, USA) and 95% sequence homology with *Mtb* oxidoreductase (MRGA423_21210) from the clinical isolate MTB423 (identified in Kerala, India). Alignment of the genes coding for these proteins show an insertion point mutation relative to Rv3368c of the reference H37Rv strain, which generated a unique C-terminus with no sequence homology with any other described protein. Phylogenetic analysis utilizing public sequence data shows that the insertion mutation is apparently a rare event. However, sera from TB patients from distinct geographical areas of the world (Peru, Vietnam, and South Africa) contain antibodies that recognize a purified recombinant C-terminus of the protein, thus suggesting a wider distribution of isolates that produce this protein.

## 1 Introduction

Urinary excretion of antigens from pathogens that cause systemic diseases, also known as “antigenuria,” has been known for decades to occur [1], [2], [3], [4], [5]. Both polysaccharides and proteins from pathogens have been detected in the urine of diseased patients [6], [7], [8], [9], [10], and in most cases, the antigenuria is not reported to be associated with renal dysfunction. Though the pathophysiology of pathogen-specific antigenuria is not completely understood, antigenuria has been used as an important premise for the development of antigen detection assays for the diagnosis of active infectious diseases. Examples of such tests are those used for the diagnosis of systemic infectious diseases caused by *Legionella pneumophilla* [11], [12], [13], *Streptococcus pneumoniae* [14], [15], [16]*, M. tuberculosis* [17], [18], [19], [20], *Leishmania donovani* complex [21], [22], [23], [24], [25], [26], *Histoplasma capsulatum* [27], [28], [29], and several others.

Previously, we have discovered and validated protein biomarkers of *M. tuberculosis* (*Mtb*) and *L. donovani* in urine of patients with active tuberculosis (TB) and visceral leishmaniasis (VL), respectively [10], [25], [26], [30], [31], [20]. In these patients, the presence of these proteins in urine correlated well with clinical manifestations of active disease.

Here, we describe the discovery and characterization of a particularly unique *Mtb* protein we have found in urine of patients with active TB. To date, the nucleotide sequence for this protein has been reported in only two clinical *Mtb* isolates. The first isolate was a widely-disseminated drug-susceptible *Mtb* strain that caused disease in New York City between 1991 and 1994 [32], [33]. This *Mtb* isolate was named the “C strain” (accession CH482375.1). More recently, the gene was also found in a clinical isolate recovered in culture from TB patients from Kerala, India [34]. This *Mtb* isolate was named “MTB423” (accession CP003234). The relationships between these two isolates, as well as their worldwide distribution, have not previously been investigated.

Here we report the characterization of this novel protein and explore immunoreactivity to the molecule in a panel of sera from patients from multiple countries who do and do not have active TB. The C-terminal half of the amino acid sequence of this protein has no known sequence match with any other microorganism. We propose that this portion of the molecule may be a useful tool for assessment of the worldwide clinical prevalence of these strains of *Mtb—*and, should prevalence warrant, for the development of novel TB diagnostic tests.

## 2 Materials and methods

### 2.1 Human samples

Two urine samples and a total of 60 serum samples from patients with pulmonary TB were evaluated in this study [all kindly provided by Foundation for Innovative New Diagnostics (FIND, Geneva, Switzerland)]. These samples were collected from patients diagnosed with TB based on a clinical course consistent with the disease and confirmatory laboratory findings (growth of *Mtb* from sputum culture). The patients providing urine were from Zimbabwe, and patients providing serum were from Peru (n = 20), Vietnam (n = 20), and South Africa (n = 20); in each of the three sets of sera, 10 of the patients had AFB smear-negative sputum, and 10 had smear-positive sputum. In addition, normal human serum (NHS) samples were obtained either from two commercial sources (ThermoFisher Scientific, Grand Island, NY and Sigma–Aldrich, St. Louis, MO) or under verbal informed consent via a sample collection protocol approved by the Forsyth Institute (n = 3). Three commercial NHS were pooled samples derived from whole blood obtained from more than 100 healthy donors per pool (ages 18–65) in the United States and processed within 24 h.

### 2.2 Mass spectroscopy analysis

Individual urine samples (15 ml) from patients with TB were concentrated using Centricon P3 (3 kDa cutoff filters) to ∼200–300 μl. Urine samples were then submitted to SDS-PAGE followed by Coomassie staining. Bands were excised from the gel and submitted for mass spectroscopy analysis at the Taplin Mass Spectrometry Facility, Harvard Medical School, Boston, MA. Excised gel bands were cut into approximately 1–2 mm wide pieces. Gel pieces were then subjected to a modified in-gel trypsin digestion procedure [35]. Gel pieces were washed and dehydrated with acetonitrile for 10 min followed by removal of acetonitrile. Pieces were then completely dried in a speed-vac. Rehydration of the gel pieces was with 50 mM ammonium bicarbonate solution containing 12.5 ng/μl modified sequencing-grade trypsin (Promega, Madison, WI) at 4 °C. After 45 min, the excess trypsin solution was removed and replaced with 50 mM ammonium bicarbonate solution to just cover the gel pieces. Samples were then placed in a 37 °C incubator overnight. Peptides were later extracted by removing the ammonium bicarbonate solution, followed by addition of a solution containing 50% acetonitrile and 1% formic acid. The extracts were then dried in a speed-vac (∼1 h). The samples were then stored at 4 °C until analysis. Samples were reconstituted in 5–10 μl of HPLC solvent A (2.5% acetonitrile, 0.1% formic acid). A nano-scale reverse-phase HPLC capillary column was created by packing 5 μm C18 spherical silica beads into a fused silica capillary (125 μm inner diameter x ∼20 cm length) with a flame-drawn tip [36]. After equilibrating the column each sample was loaded via a Famos auto sampler (LC Packings, San Francisco CA) onto the column. A gradient was formed and peptides were eluted with increasing concentrations of solvent B (97.5% acetonitrile, 0.1% formic acid). Eluted peptides were subjected to electrospray ionization and then entered into an LTQ Velos ion-trap mass spectrometer (ThermoFisher, San Jose, CA). Peptides were then fragmented to produce a tandem mass spectrum of specific fragment ions for each peptide. Peptide sequences (and hence protein identity) were determined by matching protein databases with the acquired fragmentation pattern by the software program, Sequest (ThermoFisher, San Jose, CA) [37].

### 2.3 Cloning of TBCG_03312 C-terminus gene, protein expression and purification of recombinant protein

The DNA sequence coding for the C-terminal half (aa 98–206) of the discovered *Mtb* protein (TBCG_03312 from the *Mtb* C strain)*,* was optimized for expression in *Escherichia coli*. The gene was synthesized by Blue Heron (Bothell, WA). To allow sub-cloning, restriction enzyme sequences Nde I and Bam HI were included at 5′ and 3′ endings, respectively, of the optimized DNA fragment. The synthetic gene was digested with the restriction enzymes and sub-cloned into a pET-14b expression vector, which was similarly digested for directional cloning. Protein in the pET-14b expression vector generates a six-residue histidine tag at the N-terminus of the molecule, which facilitates purification by affinity on QIAexpress® Ni-NTA agarose matrix (Qiagen, Valencia, CA) as described [38].

### 2.4 Identifying the prevalence of the TBCG_03312 protein in public *Mtb* sequence data

We downloaded shotgun sequence files from 5310 *Mtb* isolates from the NCBI sequence read archive described by Manson et al. [39]. Each pair of fastq sequence files was processed in the following bioinformatics pipeline to generate a list of variant calls: (*a*) the fastq format was confirmed using fastQValidator v 0.1.1 (https://genome.sph.umich.edu/wiki/FastQValidator); (*b*) Prinseq v 0.20.4 was used to trim reads at a quality threshold less than 20 [40]; (*c*) kraken v 0.10.5 was used to confirm that >90% of reads match *Mtb* complex taxonomic classification [41]; (*d*) trimmed reads were aligned to the H37Rv reference genome with bwa mem v 0.7.11 [42]; (*e*) samtools v 1.5 was used to calculate coverage and sequences with <95% H37Rv coverage at 10x or more were discarded [43]; (*f*) duplicate reads were removed using Picard v 2.0.1 (https://github.com/broadinstitute/picard); and (*g*) variant calling was performed after duplicate removal using Pilon [44]. We then identified all variants in this 5310 isolate set that occurred between the H37Rv coordinates 3,780,335 and 3,780,979 corresponding to the possible oxidoreductase gene Rv3368c. In the seven genomes in which we identified an insertion, we downloaded the assembly draft protein.gff file from NCBI and preformed protein-blast using the TBCG_03312 peptide. To query public finished genomes for the TBCG_03312 protein and its fragments, we used the NCBI protein and nucleotide BLAST functions.

### 2.5 Phylogenetic classification of public *Mtb* genomes

We used MUMmer [45] v 3.0 to compare the H37Rv, C strain and MTB423 finished genomes that were downloaded in fasta format from the NCBI genome database. We identified any variants overlapping with a reference set of variants from 78 phylogenetically typed genomes described by Sekizuka et al. [46] and used the concatenated set of variants as a multiple sequence alignment to build a neighbor joining phylogeny. The query strain lineage was identified as the same as the lineage of the closest reference genome on the phylogeny. For the 5310 shotgun sequences, we applied the Coll et al. [47] 67 SNP barcode to classify lineage using variants with a Pilon filter designation of PASS.

### 2.6 ELISA

ELISA was performed using standard protocols. Briefly, maxisorp surface ELISA plates (Nalge Nunc International) were coated with 50 μl of 200-ng antigen in carbonate-bicarbonate buffer/well and incubated at 4 °C overnight. Wells were aspirated and then blocked with PBS–1% bovine serum albumin at 250 μl/well at room temperature for 2 h. The blocking reagent was aspirated, and the plates were washed 5 times with PBS 0.1% Tween 20 plus 0.01% benzalkonium chloride. One hundred μl of human serum diluted in PBS at 1/100 was added per well. Samples were incubated at room temperature for 60 min. The plates were washed and 50 μl of protein A-horseradish peroxidase conjugate at a 1:20,000 dilution in PBS was added per well and incubated for 30 min at room temperature. The conjugate was aspirated, and the plates were washed. One hundred microliters of tetramethyl benzidine substrate (Kirkegaard & Perry Laboratories)/well was added and incubated for 15 min at room temperature, and the reaction was stopped with 100 μl of 1 N H2SO4/well. The plates were then read at 450 nm using an ELISA reader (ELX 808; Bio-TEK Instruments, Inc.). The cutoff for the assays was the mean of the 6 normal human serum samples plus three standard deviations of the mean.

## 3 Results

### 3.1 Identification of a unique *Mtb* protein in the urine of patients with active pulmonary TB

**Fig. 1:**
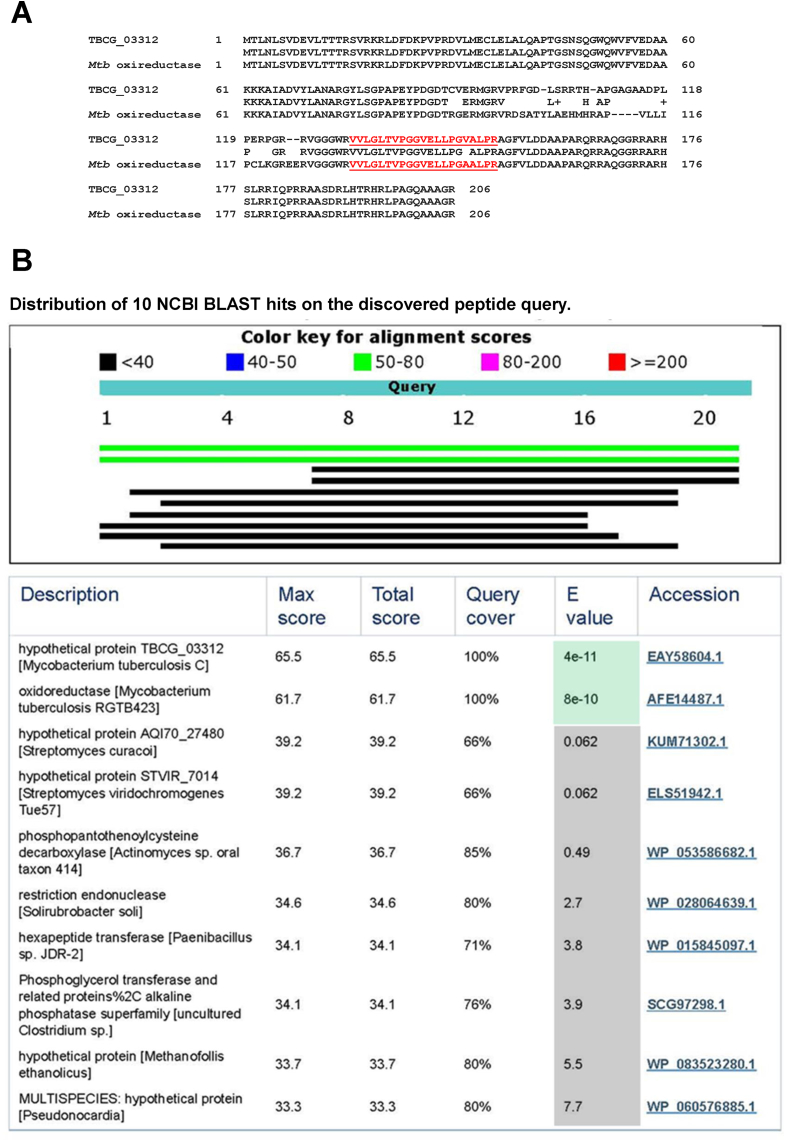
**Amino acid sequence of TBCG_03312 and *Mtb* oxireductase proteins and position of the peptide VVLGLTVPGGVELLPGVALPR (red and underlined) discovered in urine of two Zimbabwean patients with TB (A). In (B) are the top 10 NCBI BLAST hits obtained for the peptide.** Note that only the two first hits have significant E values.

Urine was collected from two patients from Zimbabwe with active, culture-confirmed pulmonary TB. Neither patient had any clinical signs, symptoms, or laboratory findings compatible with renal or urinary tract abnormalities. These criteria were important to rule out renal TB in these patients and therefore to support the proposed lung (but not kidney) origin of the *Mtb* antigens present in the patients' urine. Neither patient was on anti-tuberculosis therapy at the time of urine collection. Individual urine samples were analyzed by mass spectrometry (Methods). This analysis generated more than 500 peptide sequences. As expected, most sequences had identical sequence homologies with human proteins. However, a protein band (MW 26–37 kDa) eluted from each of the two SDS-PAGE gels (one gel for each patient's urine sample) contained one non-human 21-mer peptide sequence (VVLGLTVPGGVELLPGVALPR) with XCorr value >4.0 (Table 1). This peptide had 100% sequence homology with the deduced sequence of the protein TBCG_03312 from the C strain of *Mtb* and 95% sequence homology with *Mtb* oxireductase from the clinical isolate MTB423. Fig. 1A shows the full-length amino acid sequence of these two proteins and highlights that the discovered peptide lies in the C-terminal half of the molecules. As specificity controls, we confirmed that MS spectra for this peptide did not match any predicted *E. coli* tryptic peptides (*E. coli* being a common urinary commensal), and analysis of MS data from similarly processed urine specimens from patients with VL (and without TB) did not yield this peptide (not shown).

**Table 1:** Summary of the mass spectroscopy data for an *Mtb* peptide derived from a parent protein (TBCG_03312) found in urine of two TB patients from Zimbabwe.

Patient numerical identification	# of peptides from parent protein found in urine	Peptide sequence found in urine	Peptide Rank Charge	Ions	XCorr	ΔCn	Peptide position in parent protein
1	1	VVLGLTVPGGVELLPGVALPR	3	29/80	4.023	0.478	132–152
2	1	VVLGLTVPGGVELLPGVALPR	3	30/80	4.338	0.430	132–152

### 3.2 The TBCG_03312 C-terminus peptide is unique

The detailed BLAST analysis of the peptide revealed that its sequence matches only those of the TBCG_03312 from the *Mtb* C strain and oxidoreductase from the clinical isolate MTB423 with E-values that were extremely high and significant, i.e., 3e-11 and 7e-10 respectively (Fig. 1B). In contrast, the next possible match of the peptide sequence was with a hypothetical protein AQI70_27480 (*Streptomyces curacoi*); the E-value for this match was 0.06, and thus of no or very low significance. Other less significant ranking matches are also depicted in Fig. 1B.

**Fig. 2:**
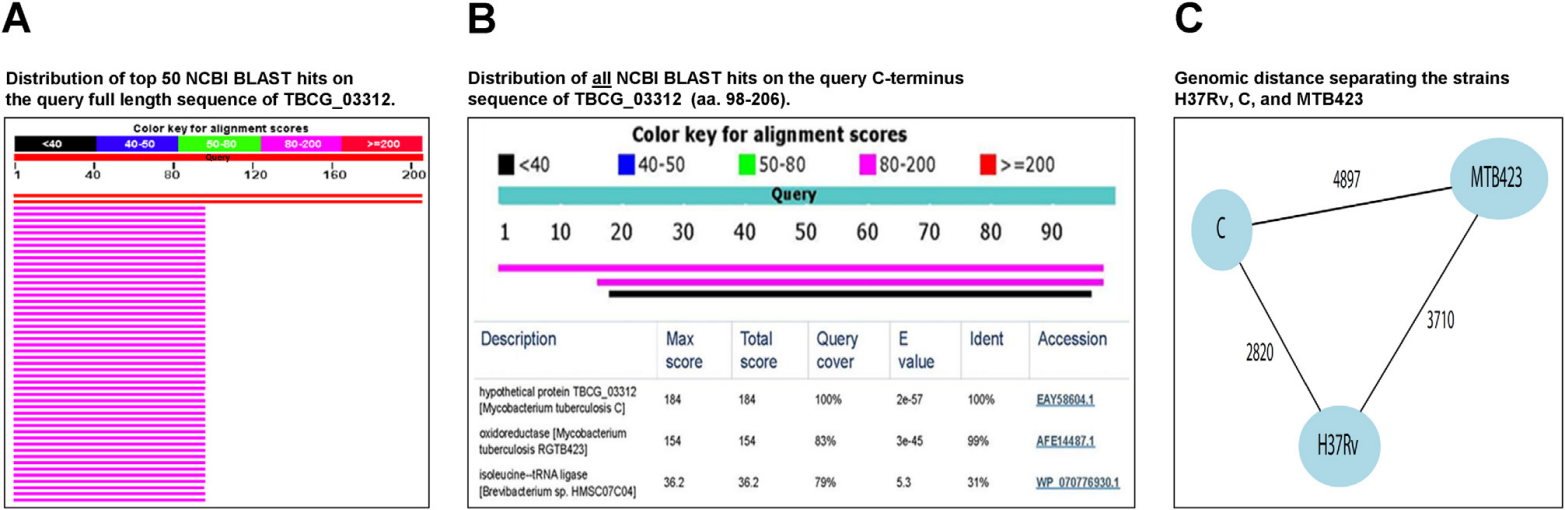
**NCBI BLAST analysis of full length and C-terminus of TBCG_03312 protein an *Mtb* oxireductase sequences**. In (A) red lines are TBCG_03312 (from *Mtb* C strain) and *Mtb* oxidoreductase (from MTB423 strain). Purple lines represent *Mtb* nitroreductase from 48 different strains of *Mtb* as well as other *Mycobacterium* species. In (B) BLAST analysis was set for Max Target Sequences of 50 and confirm that only one significant matching hit was found (oxireductase from RGTB423). Note that the E value for isoleucine-tRNA ligase from *Brevibacterium* sp is not significant. (C) represents a schematic illustration of the genomic distance separating the strains H37Rv, C, and MTB423. Distance in number of single nucleotide substitutions as obtained from a MUMmer comparison of the genomes (see Methods).

The BLAST analysis also revealed that the amino acid sequence of the N-terminal half (aa 1–97) of the donor proteins TBCG_03312 and oxidoreductase (from MTB423) is ubiquitously distributed among the genus *Mycobacterium* (Fig. 2A) and has 100% match with *Mtb* nitroreductase (Rv3368c protein). In contrast the C-terminus (aa 98–206) of the TBCG_03312 molecule, which contains the peptide VVLGLTVPGGVELLPGVALPR, is unique to the donor proteins TBCG_03312 and to oxidoreductase (from MTB423). Fig. 2B illustrates that the amino acid sequence of TBCG_03312 protein is very closely related to that of *Mtb* oxidoreductase, with an extremely high E value (3e-45) [the E value for TBCG_03312 itself is higher (2e-57) as expected because this sequence was used for the BLAST analysis]. Even with the BLAST analysis set for Max Target Sequences of 50, only one additional sequence was detected (isoleucine--tRNA ligase of *Brevibacterium* sp); however, the E value (5.3) for this alignment is not significant.

**Fig. 3:**
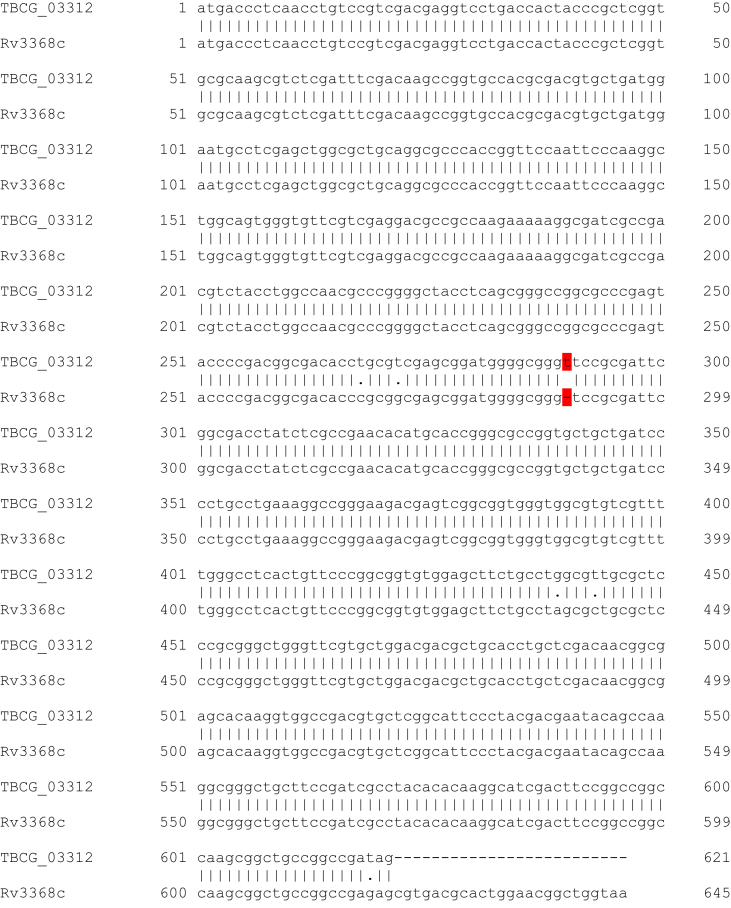
**Genomic alignment between *TBCG_03312* and *Rv3386c* loci.** The site of the insertion point mutation in *TBCG_03312* that resulted in a frame shift is highlighted in red.

Phylogenetic analysis (Fig. 2C) confirmed that the *Mtb* C strain belongs to lineage 4, specifically to sublineage 4.1.1, and is separated by 2820 single nucleotide substitutions from the Lineage 4 H37Rv reference genome. Although the C strain and MTB 423 proteome are both predicted to contain the VVLGLTVPGGVELLPGVALPR peptide they were distantly related on a genomic scale, with MTB423 belonging to lineage 1.2.2, and separated from the C-strain by 4897 single nucleotide substitutions. Finally, the gene alignment for *TBCG_03312* (C strain) and *MRGA423_21210* (MTB423) with *Rv3368c* (H37Rv) genes shows an insertion point mutation in both genes relative to *Rv3368c*. Thymine at position 289 in *TBCG_03312*, and adenine at position 371 in *MRGA423_21210* resulted in a frameshift encoding the unique TBCG_03312 C-terminus sequence (Fig. 3).

In examining 5310 public *Mtb* shotgun sequences we found an overall low level of variation in the Rv3368c H37Rv region. We found 13 different single nucleotide substitutions in 142 isolates out of the 5310 examined. The most common was a cytosine to thymine substitution at H37Rv coordinate 3780542; it occurred in 97 isolates, all of which belonged to lineage 4.4.1. There was no convergent evolution noted of these substitutions, with each restricted to a specific *Mtb* sublineage (Table 2). We also found 7 insertions putative large sequence polymorphisms all occurring at or before Rv3368c coordinate 289 (genomic coordinate≥3780623). Three of these events were an IS6110 insertion at coordinate 3780917 and occurred in 3 isolates from lineage 4.4.1.1 that were isolated from South African patients. Examination of the NCBI assembly of these genomes including the draft annotation showed no high confidence match for the TBCG_03312 C-terminus.

**Table 2:** Genetic variation in the Rv3368c gene found in 5310 public shot gun sequence [39].

H37Rv Coordinate	Change	Lineage	Number of isolates from lineage with variant	Total number of isolates from lineage studied
3780738	G > A	4.8	1	100
3780715	T > C	5	1	2
3780885	A > G	7	1	1
3780652	G > A	1.1.1	1	1
3780734	G > A	1.1.3	6	29
3780782	G > A	1.2.2	23	62
3780506	C > A	2.2.1	4	907
3780962	G > A	2.2.2	2	104
3780836	G > C	4.1	1	51
3780776	G > A	4.1.2	1	41
3780727	G > A	4.2.1	3	143
3780594	C > T	4.3.3	1	326
3780542	C > T	4.4.1	97	105
3780917	IS6110 insertion	4.4.1.1	3	100
3780647	Putative duplication	2.2.1	1	907
3780739	Putative duplication	2.2.1	1	907
3780966	Putative duplication	2.2.1	1	907
3780971	Putative duplication	2.2.1	1	907

Of the 5310 examined isolates, only 83 were classified as belonging to lineage 4.1.1, and 62 to lineage 1.2.2, i.e. predicted to be phylogenetically close to C-strain and MTB423 respectively. There were no nucleotide variants found in the 83 lineage 4.1.1 genomes, and only a single nucleotide substitution 3780782 G>A was found in 23/62 lineage 1.2.2 isolates.

### 3.3 Gene cloning and protein expression/purification of TBCG_03312 C-terminus

**Fig. 4:**
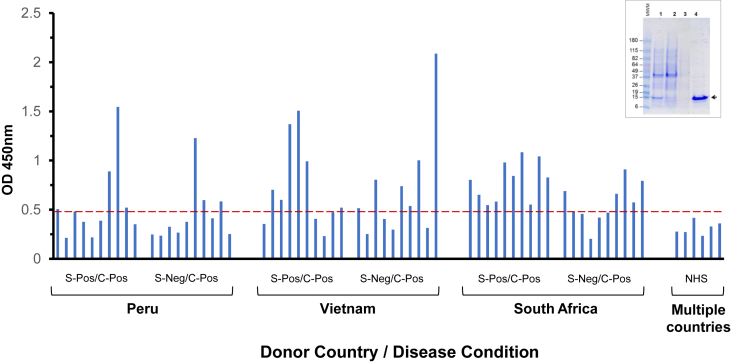
**Recognition of recombinant** TBCG_03312 **C-terminus by sera from TB patients from Peru, Vietnam, and South Africa.** Sera were tested by conventional ELISA using the purified C-terminus of TBCG_03312 [inset; Lane 1, IPTG-induced *E. coli* culture; Lane 2, flow through of IPTG-induced *E. coli* culture; Lane 3, wash; Lane 4, purified TBCG_03312 C-terminus protein (arrow)]. Dotted red line in the graph represents the mean of the OD obtained for six normal human serum (NHS) samples (Methods) plus 3 SD of the mean. S-pos, smear positive; C-pos, culture positive.

A codon-optimized synthetic gene coding for the C-terminal sequence of TBCG_03312 (aa 98–206) was obtained and sub-cloned into pET-14b expression vector. The gene was induced in *E. coli* host cells and recombinant protein was purified using a Ni-NTA agarose resin. Purity was assessed by SDS-PAGE with Coomassie blue staining. As illustrated in a single band of the expected MW (15 kDa) was obtained, indicating a high degree of purity of the recombinant TBCG_03312 C-terminus (Fig. 4).

### 3.4 Recognition of TBCG_03312 C-terminus by sera of TB patients and controls

To evaluate the possible recognition of TBCG_03312 C-terminus by antibodies from patients with TB, sera were obtained from patients with active TB living in three different geographical areas where the disease is endemic. Control sera were either commercial healthy human serum pools each derived from whole blood from more than 100 US healthy donors (n = 3) or sera obtained from individual healthy donors from Brazil (n = 3). TB patients were from Peru (n = 20); Vietnam (n = 20); and South Africa (n = 20). Recognition of TBCG_03312 C-terminus was evaluated by conventional ELISA. The results (Fig. 4) show that none of the healthy control sera reacted with TBCG_03312 C-terminus at the tested serum dilution (1/100). In contrast, the TBCG_03312 C-terminus was recognized by approximately 35%, 60%, and 75% of the sera from patients with TB from Peru, Vietnam, and South Africa, respectively (no obvious difference in serum reactivity was observed between the smear-positive and smear-negative TB patients). These results suggest both that the TBCG_03312 C-terminus is immunogenic during infection and that *Mtb* strains carrying this unique peptide (like strain C and/or MTB423) might have a more worldwide distribution than previously thought.

## 4 Discussion

In search of specific molecular markers for TB diagnostics development we serendipitously found an *Mtb* polypeptide sequence that seems to be uniquely associated with defined clinical isolate of this pathogen. The peptide sequence was found in the urines of two TB patients from Zimbabwe. The BLAST analysis of the peptide sequence matched the protein sequence (TBCG_03312) of a distinct clinical isolate of *Mtb* that, based on limited data in the literature, would seem to be rare. This strain, a drug susceptible *Mtb* called “C strain,” was described as the etiological agent of a large proportion of new TB cases occurring in New York City between 1991 and 1994 [32], [33]. Our BLAST analysis also identified a closely-related peptide (95% homology) within a more recently-reported clinical isolate of *Mtb* (MTB423) from Kerala, South India [34]. The discovered peptide initially seemed to be unique to these two strains, showing no sequence homology with any other *Mtb* strain or with any other member of the *Mycobacterium* genus by BLAST analysis. However, our observations from serologic testing suggest that *Mtb* strains harboring the gene coding for TBCG_03312 may be widely distributed; in addition to discovery of the peptide in the urine of TB patients from Zimbabwe, sera from TB patients from countries representing 3 different regions of the world (Peru, Vietnam, and South Africa) contain antibodies that recognize the C-terminus of this unique molecule. We recognize that one limitation of our study is that we were unable to test sera from currently healthy patients (with and without latent TB) from Zimbabwe/Peru/Vietnam/South Africa; it would be helpful to know whether individuals from these areas with a history of either active or latent TB would also have reactive sera, indicating that they too had been exposed to isolates producing this peptide. While we do not know the country of origin, TB history, or BCG history of the donors contributing to the pooled commercial sera we used as negative controls, we did observe that sera from 3 healthy individuals from Brazil (all BCG-vaccinated) did not react with the TBCG_03312 molecule. Given the apparently wide geographic distribution and high frequency of TB patients with serologic reactivity to the C-terminus of TBCG_03312, suggesting that *Mtb* isolates producing this unique molecule are actually widely distributed, this molecule might also be an attractive candidate for development of a urine antigen detection assay [20].

At this point two epidemiologically and phylogenetically distinct clinical isolates of *Mtb* appear to have the genetic code for the unique TBCG_03312 C-terminus (*Mtb* C strain and MTB423); our serological findings cannot pinpoint the actual *Mtb* strain that caused TB in the patients evaluated in our study. However, given that the unique sequence of the TBCG_03312 C-terminus was generated by an insertion mutation in the Rv3386c gene, and the rarity of these insertion mutations in other examined public sequence data, it is possible that the strains that infected the patients who provided the sera we evaluated are related to either C strain or MTB423.

Although our search through public sequence data revealed a low level of variation in the Rv3368c gene and notably a lack of single base insertions in lineages phylogenetically close to the C-strain and to MTB423, the results are limited by the lack of finished or complete sequence data available for *Mtb* lineages 1 and 4.1.1 in the public domain. It is worth noting that whole genome sequencing efforts in TB have to-date not been systematic or designed to be accurately representative of the burden of different TB lineages, and biased towards cases from developed countries and with drug resistance [48].

Despite using a bioinformatics pipeline that incorporates read assembly and increases the sensitivity for predicting insertions, deletions and sequence polymorphisms, it is possible that our pipeline was conservative and that we missed relevant variation that could explain the ELISA results. Further, in our analysis of the few genomes we found with insertions we relied on a draft automated protein annotation that may not be reliable. Therefore, further studies of finished genomes from lineage 4.1.1 and lineage 1 are still needed to definitively interpret these information in conjunction with serological data.

Moreover, proteome analysis of *Mtb* C and/or MTB423 strains, will be required to assess and confirm the potential utility of diagnostics development based on the TBCG_03312 C-terminus peptide. These evaluations will include: first, the molecular detection (e.g., by mass spectroscopy or RT-PRC) of the unique C-terminus polypeptide in cultures of *Mtb* C strain, MTB423 and/or other isolates; second, production of specific antibodies to the polypeptide and assemble of a sensitive capture ELISA followed by a clinical investigation to determine if such an antigen detection assay could help the diagnosis of TB.

## Conflict of interest

None of the authors has any financial conflict of interest.
